# The Contribution of Online Peer-to-Peer Communication Among Patients With Adrenal Disease to Patient-Centered Care

**DOI:** 10.2196/jmir.3869

**Published:** 2015-02-25

**Authors:** Dirkjan Kauw, Han Repping-Wuts, Alida Noordzij, Nike Stikkelbroeck, Ad Hermus, Marjan Faber

**Affiliations:** ^1^Radboud University Medical CenterDepartment of Internal Medicine, Division EndocrinologyNijmegenNetherlands; ^2^Dutch Adrenal SocietyNijkerkNetherlands; ^3^Radboud University Medical CenterInstitute for Quality of HealthcareNijmegenNetherlands

**Keywords:** online forum, self care, Cushing’s syndrome, Addison’s disease

## Abstract

**Background:**

Addison’s disease and Cushing’s syndrome are rare. The Dutch Adrenal Society offers an online forum for Dutch adrenal patients to meet and communicate. However, little is known about the added value such a forum has for the delivery of patient-centered care.

**Objective:**

Our aim was to analyze the purposes of online patient-to-patient forum conversations, within the context of patient-centered care.

**Methods:**

For this study a consecutive sample of 300 questions (“threads”) from the past 3.5 years was selected from the forum. The content of these patient-driven questions was analyzed based on the dimensions of patient-centeredness of the Picker Institute. This analysis was performed using ATLAS.ti.

**Results:**

From the 390 questions analyzed, 80.8% (N=315) were intended to gain more information about the disease, the treatment, and to verify if other patients had similar complaints. To a much lesser extent (38/390, 9.7%), questions expressed a call for emotional support. Patients answered primarily by giving practical tips to fellow patients and to share their own experiences.

**Conclusions:**

On an online patient forum for Cushing’s syndrome and Addison’s disease, patients appear to primarily gain knowledge and, to a lesser extent, emotional support from their peers. This experience-based knowledge has become a very important information source. As such, patients can make a substantial contribution to the creation of patient-centered care if this knowledge is integrated into the care provided by health care professionals.

## Introduction

Addison’s disease and Cushing’s syndrome are rare disorders, characterized by abnormal secretion of adrenal hormones. Patients with Addison’s disease, but also many patients with Cushing’s syndrome after treatment, require long-term substitutional therapy [1]. It affects their lives dramatically, either physically, emotionally, or both [2-6]. Both conditions have a significant impact on patients’ quality of life. Patients treated for Cushing’s syndrome, for example, show increased levels of anxiety and symptoms of depression [2,4-6].

With long-term substitutional therapy, a certain amount of self-care and knowledge is expected from the patient. The level of knowledge in patients and the ability to react adequately in critical situations is often insufficient [7]. Not recognizing predictive signs of an adrenal crisis or not knowing when to increase the dosage of the medication is detrimental for these patient groups. This lack of knowledge sometimes even leads to hospitalizations and life-threatening events [8,9]. Patients with substitutional therapy are advised to carry a medicine passport with them and an “SOS medallion”, but not every patient follows this advice [8]. Group education and other educational programs can increase the quality of self-management and the use of self-management tools like the SOS medallion and the medicine card in adrenal patients [7,10].

To support self-management effectively, patient-centeredness should be a cornerstone of the health care delivery model [11]. Patient-centeredness is usually considered to be merely a task for health care providers. However, in the past decade the role of peer-to-peer support by means of online forums has gained tremendous ground, offering the potential to support self-management [12]. Online support groups can be described as “weak tie” networks, as the majority of the users only communicate through online messages. The people on the forum can have daily contact but are not necessarily close [13]. Patients use online forums to post messages and ask questions. Previous forum analyses revealed that patients primarily retrieve two main types of support from online support groups: experiential information and emotional support [14-17]. This type of online support cannot be acquired in the consultation with the doctor or taught in educational programs, but it can contribute to aspects of patient-centered care [18].

The aim of this study was to analyze the purpose of peer-to-peer communication, that is, what is the communication being used for, within the context of patient-centered care. The study is meant to show the potential contribution of online peer-to-peer communication to the delivery of patient-centered care.

## Methods

### Data

Conversation data were obtained from the online forum moderated by the Dutch Adrenal Society (NVACP). This is a member-only forum, requiring user login, except for the subforum “Public”, which is open to everyone. This forum is moderated by a team of volunteers, and rules are stated on the website to make sure the forum is used appropriately. For example, if a user wants to post medical information, references are demanded. If the requirements are not met or rules are violated, the message gets deleted or modified. There were 1210 registered members of the NVACP on January 1, 2014. For the purpose of our study, we created a static database to prevent any changes that could be made to the posts on the forum by the members or the administrators of the forum. We did this by downloading the posts into an Excel file. We used a sample of 300 consecutive questions (“threads”) in this study, consisting of 100 threads from three subforums each—Public, Addison, and Cushing—as these were the three most active subforums. For every thread we included, we also analyzed every first answer that was posted. The oldest thread dated from June 2010, and the most recent thread dated from January 2014.

To prevent any violation of privacy and to protect the identity of the members of the forum, no names of individuals or personal information were included or used in the analysis. A message was posted on the general subforum on the site on January 14, 2014, informing the forum members about this study and also that their identities and their personal information would not be used in the study. This procedure was presented to the local medical ethics committee and approved.

### Analysis

We performed a deductive qualitative framework analysis for the 300 selected threads [19]. As a theoretically informed framework, the eight principles of patient-centered care of Picker guided the analysis: access to care, coordination and integration of care, emotional support, involvement of family and friends, information, communication and education, physical comfort, respect for patients’ values, and transition and continuity of care [20]. Every thread was labeled with one or more of the principles. In case one thread contained multiple subquestions, all subquestions were included.

For analyzing the answers to the questions, we used open coding [19]. Driven by the list of answers, we created a list of response themes that represented the aim of the answer. This list was created in an iterative manner, after reading through the answers multiple times.

Two researchers independently analyzed the data (DK, HR-W). Both had to agree on the principle that was selected for each question and theme for the answer. If both researchers did not agree, a third researcher (MF) joined the discussion until consensus was reached. We used the program ATLAS.ti for coding and supporting the analysis.

If questions were posted twice on the same subforum, for example due to a mistake by the poster or a technical error, these duplicate questions were analyzed only once to prevent biased results. If questions were posted twice but on different subforums, this was most likely done deliberately to get more views and replies to the question. These duplicate questions were therefore included in the analysis.

## Results

The time span in which 100 threads were posted differed per subforum. The Cushing subforum was less active than the Addison and Public subforum. The 100 threads from the Cushing subforum were created in a time span of 3 years, whereas the 100 Public and Addison threads were created in a time span of just over 1 year.

###  Questions

The 300 threads included a total of 390 separate questions. Taking the three forums together, 80.8% (315/390) of questions fitted into one Picker principle, that is, “Information, communication, and education”. Because of the large number of issues, we created subthemes: treatment, the facility, medication, practical questions, similar complaints, suspected disease, and the disease itself. On every subforum, “similar complaints” (26.3%, 23.5%, 26.9% for Public, Addison, and Cushing respectively) and “question about the disease” (15.3%, 18.5%, 20.1%) were the most common used subthemes. “Emotional support” was the second most important principle, represented in 38 threads (9.7%), whereas no questions related to the principles “physical comfort” and “transition and continuity of care”. A small number of 17 threads did not fit any of the principles and were therefore labeled as “Miscellaneous” (4.6%) (see Table 1). The “Miscellaneous” category covered threads about the forum itself and about meetings of the NVACP.

**Table 1:** Total amount of questions, categorized per Picker principle.

Picker principles		Public (N=137), n (%)	Addison (N=119), n (%)	Cushing (N=134), n (%)	Total (N=390), n (%)
Access to care		3 (2.2)	1 (1.0)	2 (1.5)	6 (2)
Coordination and integration of care		0 (0.0)	1 (1.0)	0 (0.0)	1 (0.0)
Emotional support		11 (8.0)	11 (9.2)	16 (11.9)	38 (9.7)
Involvement of family and friends		2 (1.5)	3 (2.5)	0 (0.0)	5 (1.3)
**Information, communication, and education**		108 (78.8)	97 (81.5)	110 (82.1)	315 (80.8)
	Question about treatment	15 (10.9)^a^	13 (10.9)	25 (18.7)^a^	53 (13.6)
	Question about the facility	4 (2.9)	3 (2.5)	7 (5.2)	14 (3.6)
	Medication	10 (7.3)	22 (18.5)^a^	14 (10.4)	46 (11.8)
	Practical questions	9 (6.6)	7 (5.9)	0 (0.0)	16 (4.1)
	Similar complaints	36 (26.3)^a^	28 (23.5)^a^	36 (26.9)^a^	100 (25.6)
	Suspected disease	13 (9.5)	2 (1.7)	1 (1.0)	16 (4.1)
	Question about the disease	21 (15.3)	22 (18.5)	27 (20.1)	70 (17.9)
Physical comfort		0 (0.0)	0 (0.0)	0 (0.0)	0 (0.0)
Respect for patients’ values		3 (2.2)	3 (2.5)	2 (1.5)	8 (2.1)
Transition and continuity of care		0 (0.0)	0 (0.0)	0 (0.0)	0 (0.0)
Miscellaneous		10 (7.3)	3 (2.5)	4 (3.0)	17 (4.6)

^a^The three most frequent question themes per subforum.

A frequent example of a question that was coded as “similar complaint” was “Does anyone have a similar experience?” Two more specific examples of “similar complaint” questions are:

I do a lot of exercise, everything gets toned, but my stomach remains swollen. I also find that I get more and more stretch marks on my stomach though not growing anymore and not gaining any weight. I would like to know who has experienced this and what can be done about it.

1 year ago I had surgery. My ACTH-producing adenoma was successfully removed. I’m still phasing out with HC and now use 10 mg. per day. Every reduction of dosage is hard. Lately I’ve noticed that I have more energy at night than during the day. My own theory is that this is because a healthy person also has low cortisol levels in the evening. I wonder if there are more people who recognize this.

On the Public and Cushing subforums, the “treatment” subtheme was also quite frequent, with respectively 11.1% of the questions (15/135) and 18.7% of the questions (25/134) from these specific subforums. On the Addison subforum, “medication” was more frequent than on the other subforums: 18.5% of questions (22/119), compared to 7.3% (10/137), and 10.4% (14/134) for the Public and Cushing subforums, respectively.

### Answers

The final list of response themes contained 12 items, and an answer could have multiple themes assigned to it. The response themes were to emphatically urge, to provide medical information, to provide practical information, a counter-question, share own experiences, be motivational, to set at ease, to support, to hint, give advice to consult the doctor, miscellaneous, and questions answered by the poster themselves. A total of 29 (7.2%) questions remained unanswered (see Table 2). Quite often, regardless of the subforum, answers were provided that related to “own experiences” (21.5%, 23.9%, 26.6% for Public, Addison, and Cushing, respectively).

**Table 2:** Total amount of answers, categorized per theme.

Goal of the answer	Public (N=130), n (%)	Addison (N=130), n (%)	Cushing (N=143), n (%)	Total (N=403), n (%)
Emphatically urge	3 (2.3)	2 (1.5)	1 (0.6)	6 (1.5)
Medical information	16 (12.3)	12 (9.70)	13 (9.1)	41 (10.2)
Practical information	3 (2.3)	4 (3.1)	2 (1.4)	9 (2.2)
Counter-question	18 (13.8)^a^	24 (18.5)^a^	15 (10.5)	57 (14.1)^a^
Own experiences	28 (21.5)^a^	31 (23.9)^a^	38 (26.6)^a^	97 (24.1)^a^
Motivational	0 (0.0)	0 (0.0)	1 (0.6)	1 (0.0)
Set at ease	1 (1.0)	0 (0.0)	0 (0.0)	1 (0.0)
Support	11 (8.5)	13 (10)	20 (14)^a^	44 (11.0)
Hint	20 (15.4)^a^	33 (25.4)^a^	28 (19.6)^a^	81 (20.1)^a^
Advice to consult the doctor	16 (12.3)	2 (1.5)	7 (4.9)	25 (6.2)
Miscellaneous	4 (3.1)	0 (0.0)	0 (0.0)	4 (0.9)
Answered by poster	2 (1.5)	3 (2.3)	3 (2.1)	8 (2.0)
Question unanswered	8 (6.2)	6 (4.6)	15 (10.5)	29 (7.2)

^a^The three most frequent answer themes per subforum.

A good example of an “own experience” answer is “I recognize it and I have the same complaints”. The following is an example of a conversation with a “similar complaint” question and an “own experience” answer:

Q: Dear people, are there people with Addison who can sleep fast at night?? Whatever I try, I always wake up in the middle of the night and then I have a lot of trouble falling asleep again. Again who oh who can help me with some good advice eg one or other substance, or dietary supplement or whatever, I would be very happy.

A: I have also tried…before and I had a dull feeling in the morning. If I’m still really busy I get out of bed and I use…(also homeopathic), walk outside with my dog as a distraction and then get back into bed. It doesn’t always work, but usually it does. Hopefully this can be useful to you. Greeting and a happy and above all a healthy 2014!

“Hint” (33/130, 25.4%; 28/143, 19.6%) and “counter-question” (24/130, 18.5%; 15/143, 10.5%) were mainly frequent on the Addison and Cushing subforums respectively. The advice to consult a doctor was more often provided in the Public subforum (16/130, 12.3%) compared to the members-only subforums of Addison (2/130; 1.5%) and Cushing (7/143; 4.9%).

## Discussion

### Principal Results

This is the first study of online peer-to-peer communication in Addison’s disease and Cushing’s syndrome patients. The forum we studied was mainly used to express informational cues, and many of these questions were answered by sharing own experiences, that is, sharing experienced-based knowledge. An online peer-to-peer communication forum has the potential to provide a meaningful, but narrowly focused, contribution to patient-centered care, as over 90% of the questions related to two of the eight Picker principles for patient-centeredness.

### Comparison With Prior Work

In the research on peer-to-peer communities, two methods are used to gain insight in these forums. The content of the forums can be analyzed in a qualitative analysis or information is gained by means of questionnaires or interviewing users of peer-to-peer forums. The first provides insight into what kind of purpose the forum has for the patients, whereas the second focuses more on the psychological and patient empowerment outcomes that patients experience.

The results of our study give us insight into the needs of Dutch patients with Addison’s disease or Cushing’s syndrome. The diseases are rare and complicated, and the number of patients is relatively low. Online these patients seek information support and, to a much lesser extent, emotional support. Multiple other patient forums, for a range of diseases, have been analyzed, and they also show the role of informational and emotional support of online forums [15,16,21]. In the NVACP forums, the balance between informational and emotional cues heavily leans towards the informational cues, which mimics the type of questions patients ask during a doctor consultation. In such a doctor-to-patient setting, emotional cues are about half as frequent compared to informational and illness-related cues [22].

Health can be positively affected by sharing experiences with peers, as described by Ziebland and Wyke [17]. Acquiring information, getting emotional support and supporting one another, describing your disease, and motivating each other to take actions as mechanisms for such positive influence were found in our results. However, evidence for positive outcomes of online support groups on health is inconclusive. In a systematic review about online support groups among cancer survivors, most studies report positive effects on psychosocial outcomes, but none of them reported significant outcomes [23].

### Online Support Groups as Contributors to Patient-Centered Care

Our study shows the potential that online peer-to-peer support has in making a contribution to patient-centered care. Apart from the contribution to the “Information, communication, and education” principle, online forums can be accessed any time of the day, every day, and from anywhere. As such, it improves “Access to care” by removing geographical and social barriers and making communication more convenient and accessible. Also, online support groups are not constrained by space, as happens online. At the same time, it eliminates many people globally that do not have access to the Internet on a daily basis or who are not able to read and write.

A very important benefit of online support groups is that patients become engaged in their care, just by gaining experiential information and receiving emotional support from their peers. The interaction between the health care professional and the patient versus the interaction between patients on online forums are therefore interesting to compare. In a consultation of a patient with a physician, the physician usually fulfils two roles: an instrumental role and an affective role. Instrumental means the information exchange between the physician and the patient, and affective means building up an emotional relationship with the patient [24]. It is not clear to what extent both roles could be displayed during online conversations. Vennik et al [18] analyzed doctor-patient conversations in online forums. Patients value doctors’ feedback differently from feedback received by peers. Peer-to-peer support usually is used as experiential information, whereas feedback by health care professionals is considered more reliable and evidence based.

Another reason for patients to seek information on forums could be because of inadequate information provision channels (eg, verbal or written folders) provided by professionals or inadequate informational content in existing materials. Van de Belt et al [25] analyzed whether the questions patients asked on an online forum could be answered with the information that was provided in the patient folders. About half of the patients’ questions could not be answered with the information from the patient folders. As a result, questions concerning medical information arise on patient forums and then can be satisfactorily answered by peers, acknowledging the experienced-based value of information from peers. Therefore, it seems likely that patient forums are perceived as useful and even indispensable to patients.

### Limitations

There are some limitations to this study. We analyzed only every first answer of every question. This excludes the remainder of the conversation. Also in this study, the posters of the messages were made anonymous. This was done to protect the identity and privacy of the posters. As a consequence, however, it excluded some interesting data for analysis. For example, we could not differentiate posters from lurkers or distinguish very active posters from one-time posters. Some patients are more active on forums than others. These active forum users who post messages regularly are called posters, and the forum users who mostly read the messages on the forum rather than posting new messages themselves are called lurkers. However, both groups seem to profit equally from peer-support via forums [26]. Also, only the 100 newest threads from the three subforums were analyzed. This excludes older threads that could still be very up to date and very active. Also the true meaning of each post could never be totally clear to the researchers, as the expression of the poster fully depends on how they formulate their posts. Finally, we did not rate the quality of the responses, that is, was the experienced-based knowledge in line with evidence-based guidelines, or was the response appropriate for the expressed cue?

### Conclusions

In conclusion, on an online patient forum for Cushing’s syndrome and Addison’s disease, patients primarily gained knowledge and emotional support from their peers. Patients are therefore able to provide a significant contribution to the creation of patient-centered care. Moreover, the questions raised in an online forum revealed unmet needs and issues that matter to patients. As such, online peer-to-peer communication is an excellent resource for improving the delivery of patient-centered care tailored to the unmet needs of patients.
